# Phylogenetic and molecular characterization of coxsackievirus A24 variant isolates from a 2010 acute hemorrhagic conjunctivitis outbreak in Guangdong, China

**DOI:** 10.1186/1743-422X-9-41

**Published:** 2012-02-15

**Authors:** Wu De, Zheng Huanying, Li Hui, Monagin Corina, Guo Xue, Liu Leng, Zeng Hanri, Fang Ling, Mo Yanling, Zhou Huiqiong, Zhang Huan, Kou Jing, Long Caiyun, Hiromu Yoshida, Ke Changwen

**Affiliations:** 1 Institute of Microbiology, Center for Disease Control and Prevention of Guangdong, No.176, Xingang Road W, Guangzhou, Guangdong, 510300, People's Republic of China; 2 Global Viral Forecasting, San Francisco, CA, 94104, USA; 3 National Institute of Infectious Diseases Gakuen, Tokyo, 2080011, Japan

**Keywords:** CA24v, AHC, Molecular epidemiology, Phylogenetic analysis, Guangdong

## Abstract

**Background:**

Acute hemorrhagic conjunctivitis is a common disease in China. As a notifiable disease, cases are registered by ophthalmologists on the AHC surveillance system. An AHC outbreak caused by CA24v was observed in Guangdong Province in 2007 by the National Disease Supervision Information Management System. Three years later, a larger outbreak occurred in Guangdong during the August-October period (2010). To characterize the outbreak and compare the genetic diversity of CA24v, which was determined to be the cause of the outbreak, the epidemiology and the molecular characterization of CA24v were analyzed in this study.

**Results:**

A total of 69,635 cases were reported in the outbreak. 73.5% of index cases originated from students, children in kindergarten and factory workers, with the ≦ 9 age group at the highest risk. The male to female ratio was 1.84:1 among 0-19 years. 56 conjunctival swabs were collected to identify the causative agent from five cities with the AHC outbreak. 30 virus strains were isolated, and two of the genomes had the highest identity values (95.8%) with CA24v genomes. Four CA24v genotypes were identified by phylogenetic analysis for the VP1 and 3C regions. CA24v which caused the outbreak belonged to genotype IV. Furthermore, full nucleotide sequences for four representative isolates in 2010 and 2007 were determined and compared. 20 aa mutations, two nt insertions and one nt deletion were observed in the open reading frame, with 5'- and 3'- UTR respectively between them.

**Conclusions:**

CA24v was determined to be the pathogen causing the outbreak and belongs to genotype IV. VP1 is more informative than 3C^Pro ^for describing molecular epidemiology and we hypothesize that accumulative mutations may have promoted the outbreak.

## Background

Acute hemorrhagic conjunctivitis (AHC) is a highly contagious infection, characterized by an abrupt onset of ocular pain, swelling of the eyelids, a foreign body sensation or irritation, epiphora, eye discharge and photophobia [1,2]. CA24v, EV70 and some additional adenovirus serotypes are the major etiological agents of AHC [3]. Outbreaks of AHC caused by CA24v were first described in Ghana in 1969 [4], with the first isolation of CA24v causing AHC reported during an outbreak in Singapore in 1970 [5]. In the past several decades, CA24v was recognized as the major causative agents of AHC outbreaks [6-8]. AHC spread to Mainland China in 1971 [9], and CA24v was identified in Hong Kong in 1975 [10].

In a previous study, nucleotide sequence variations of the 3C^Pro ^regions of the CA24v genome were compared by using isolates from various regions of the world. Phylogenetic analysis revealed that CA24v appeared at one focal point in Asia around 1963 [11]. Numerous AHC epidemics have occurred since 1969. A high degree of infectivity of CA24v to the human conjunctiva was conferred by mutations accumulating in the RNA viral genome causing expanded epidemics [12]. Four genotypes of the CA24v were described and identified by phylogenetic analysis of the 3C^Pro ^and VP1 regions of the genome [13]. Recently, a large epidemic by CA24v was documented in China in 2007. Phylogenetic analyses revealed these isolates were located in the same cluster, and have the closest relationship to the 2005 Singapore isolates [8,14-16].

Another larger AHC outbreak caused by CA24v was observed in 2010 in Guangdong, China. In this report, we briefly describe the epidemiology of the outbreak. In addition, in order to characterize the viral genome of the CA24v isolates in 2010, the 3C^Pro ^and VP1 region of the viral genome, as well as complete nucleotide sequences, were determined and phylogenetically analyzed for isolates collected from AHC patients.

## Results

### The outbreak

**Figure 1:**
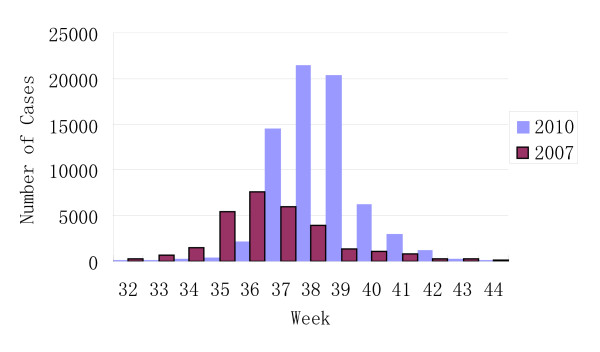
**Weekly case numbers of acute hemorrhagic conjunctivitis reported by the disease surveillance network in Guangdong between July and October of 2007 and 2010**.

As AHC is a notifiable infectious disease in China, all cases diagnosed by physicians were registered in the NDSIMS. Surveillance data showed the number of AHC cases noticeably began to increase from the 36th week and reached a peak of over 20,000 cases in the 38th week in 2010 in Guangdong province. The number of AHC cases returned to a baseline of around 200 cases in the 43rd week. Although in a previous report [8], the peak of the outbreak was identified to be during the 35th-37th weeks, the peak of the 2010 outbreak was in fact between the 37-39th weeks (Figure 1). A total of 72,181 cases were reported in 2010 in Guangdong. In this outbreak, 69,635 AHC cases (74.9 cases/100,000 population) were reported between Sep 1 and Oct 31, 2010. The number of AHC cases during 32~44 weeks was on average 195-fold (69,759/358 cases) and 2.5-fold (69,759/28,381 cases) higher than 2009 and the outbreak of 2007 respectively. Among these cases, 56% percent were male. The male to female ratio was 1.84:1 (1.62:1,2007) among 0 ~ 19 years, and 1.33:1 (1.44:1, 2007) among 0 ~ 49 years, but the ratio was 0.85:1 (0.79:1, 2007) among the ≧ 50 age groups.

**Figure 2:**
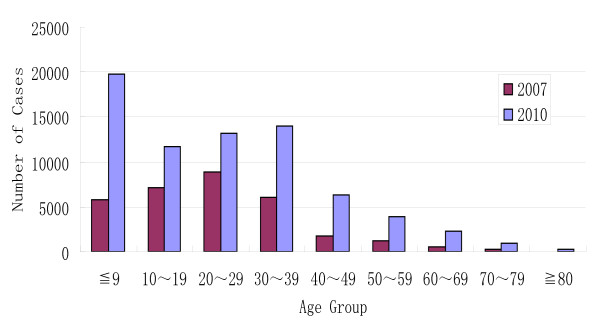
**Age distribution of acute hemorrhagic conjunctivitis cases confirmed by ophthalmologists in 2007 and 2010**.

Although cases were reported throughout the province, cases were most frequently reported in 5 cities; Heyuan, Guagnzhou, Zhaoqing, Foshan and Jianmen. 23.9% of cases were students, followed in frequency by factory workers (22.8%) and children in kindergartens (16.8%). Case-patients occurred in all age groups, and most of them (81.1%) were < 40 years of age, similar to the 2007 outbreak (87.8%). Two age peaks occurred in the ≦ 9 and the 30 ~ 39 age group. The population at highest risk was ≦ 9 years of age (27.3%) (Figure 2). Only one age peak was observed in the 20~29 age group in the 2007 outbreak (Figure 2).

### Samples collection and virus isolation

A total of 56 conjunctival swabs were collected from 56 AHC patients aged 2 months -56 years during the acute phase (1-5 days after onset of symptoms). These cases were comprised of 27 males and 29 females, mainly from students at kindergartens and schools, and factory workers in 5 cities of the Guangdong region. These samples were collected from Guangzhou (10 samples), Heyuan (16 samples), Jiangmen (8 samples), Zhaoqing (10 samples) and Foshan (12 samples) on the 14th and 30th of September. Virus isolation was performed in HEp-2 cell line. CPE were clearly observed on the HEp-2 cell line for 30 of the 56 samples.

### RT-PCR tests

The 3, 4, 3, 3 and 3 representatives from Guangzhou, Heyuan, Jiangmen, Zhaoqing and Foshan (respectively) were subjected to one step RT-PCR with 3C-1, 3C-2 and CVA24v-S, and CVA24v-A specific primers encoding the 3C^Pro ^and VP1 region on the CA24v genome. 32 special RT-PCR products from 16 isolates were observed clearly in 1.0% agarose gels. In addition, we used the four representatives isolated from Guagnzhou and Heyuan in 2010 and 2007 and performed one-step RT-PCR with eight other special primers. 32 special bands from four isolates were observed in 1.0% agarose gels.

### Sequence comparison and phylogenetic analysis of the 3C^pro ^and VP1 regions

As there were few CA24v full genome sequences available in GenBank, and our main objective was to not only describe the epidemiology of the 2010 outbreak, but also to compare the Guangdong sequences with those from other AHC outbreaks, we carried out a phylogenetic analysis in the 3C^Pro ^and VP1 regions which were available from previous studies.

**Figure 3:**
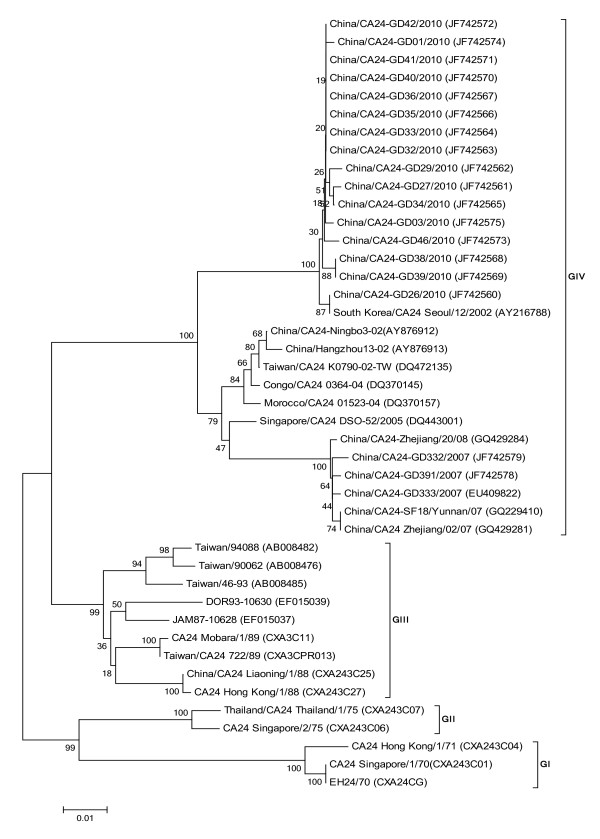
**Phylogenetic analysis based on the partial 3C3C^Pro ^(549 nucleotides)**. The nucleotide sequences were analyzed using a MEGA5.0 package. Four topologies were inferred by Neighbor-joining cluster analysis with the bootstrap option. The numbers at the branching points are bootstrap values estimated with 1,000 pseudo-replicate data. Four genotypes (G I-IV) are indicated. The last numbers of each strain name indicate the year of isolation. The locations are shown to the right of the strain name.

16 isolates from 2010 with a 549 bp size fragment on the 3C^Pro ^region were used for analysis with 27 CA24v strains from each genotype that had caused epidemics of AHC. The 3C^Pro ^nucleotide sequence identity values among 43 strains had a range of 85.4-100% (data not shown), corresponding to a 95.1-100% of amino acid identity values on the 3C^Pro ^protein. Four genotypes were chronologically clustered in the tree (Figure 3). Genotype I (G I) contained early isolates from Singapore, Hong Kong and the CA24v prototype obtained in 1970-1971. 98.5-100% identity values in the nucleotide sequence were observed. Genotype II (G II) was composed of isolates from Singapore and Thailand obtained in 1975, with 98.7% identity values. Eight isolates from 1988 to 1994 were from South-East Asia and the Americas, and were included in genotype III (G III), with 88.5-99.8% identity values in their nucleotide sequences. The other isolates from 2002 to 2010 were located in genotype IV (G IV), with 90.3-100% identity values. Guangdong strains isolated in the 2007 and 2010 outbreaks belonged to G-IV, but were located in different clusters of the Singapore strains in 2005 and South Korea strains in 2007.

**Figure 4:**
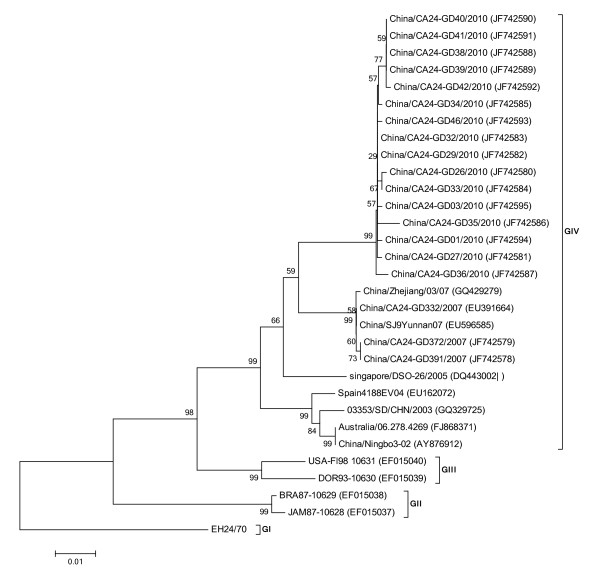
**Phylogenetic analysis based on the entire VP1 protease (915 nucleotides)**. The nucleotide sequences were analyzed using a MEGA5.0 package. Four topologies were inferred by Neighbor-joining cluster analysis with the bootstrap option. The numbers at the branching points are bootstrap values estimated with 1,000 pseudo-replicate data. Four genotypes (G I-IV) are indicated. The last numbers of each strain name indicate the year of isolation. The locations are shown to the right of the strain name.

To elucidate the nucleotide variation in the VP1 region of strains isolated in Guangdong in 2010, 16 virus strains from 2010, three from 2007 (JF742580-JF742595, JF742578, JF742579, EU391644) and 12 strains from each genotype and different geographical locations were selected for the analysis of 915 bp of the VP1 region. Percent of nucleotide identity values ranged from 85.6 to 100% on the VP1 region, corresponding to a 97.4-100% of amino acid identity values on the VP1 protein. Phylogenetic analysis of the CA24v strains showed 4 distinct genotypes: group I, consisting of one prototype strain EH24/70 (D90457); group II, consisting of BRA87-10629 (EF015038) and JAM87-10628 (EF015037) strains from 1987, with 100% identity values; group III, consisting of USA-Fl98 10631 (EF015040) and DOR93-10630 (EF015039) strains from 1993 to 1998, with 98% identity values; and group IV, consisting of more diverse strains and including some AHC isolates from 2003 to 2010, with 94-100% identity values (Figure 4). From the phylogenetic analysis, the Guangdong 2010 and 2007 isolates were included into group IV, with CA24v isolates associated with AHC epidemics isolated in China, Korea, Singapore, Spain and Australia since the year 2000, which had a close relationship with singapore/DSO-26/2005 (DQ443002).

### Genome sequence comparison and analysis

Four complete genome sequences of CA24v, which were isolated from two large outbreaks in 2007 and 2010, were obtained from the overlapping amplicons using 10 primer sets total, which were also isolated from two outbreaks in 2007 and 2010 respectively.

**Figure 5:**
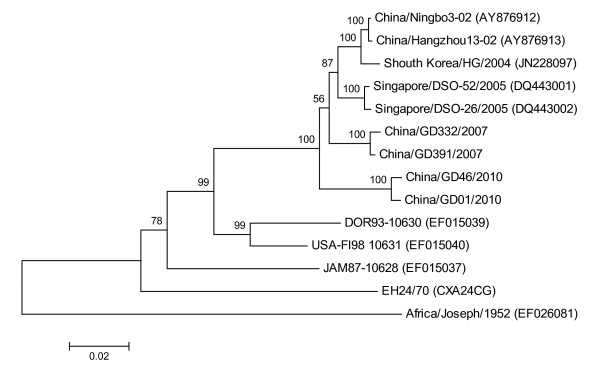
**Phylogenetic analysis based on the complete genome (7,460 nucleotides)**. The nucleotide sequences were analyzed using a MEGA5.0 package. The phylogenetic tree was constructed by Neighbor-joining cluster analysis with the bootstrap option. The numbers at the branching points are bootstrap values estimated with 1,000 pseudo-replicate data. The last numbers of each strain name indicate the year of isolation. The locations are shown to the right of the strain name.

Their genome sizes had lengths of 7,463 bp with 750 nt at the 5'-UTR, and a short, 69-nt sequence at the 3'-UTR, with G + C contents of 45.2% (2010) and 45.6% (2007) respectively. The polyprotein precursor is encoded by a long open reading frame of 6642 nt, corresponding to 2214 aa. Phylogenetic analysis was performed using the complete genome of four strains from this study and 10 worldwide strains from different AHC outbreak events and regions in GenBank. The genome sequence identity values among 14 strains were 80.3-99.3% (data not shown). Two genomes from 2010 had a range of 95.7-95.8% identity values with two 2007 genomes, and 95.6% identity values with Singapore/DSO-2/2005 (DQ443001) at the nucleotide level. Phylogenetic analysis showed the four genome isolates were located in a large cluster, and were separated into two sub small branch clusters (Figure 5).

Twenty common amino acid residue changes, two nucleotides insertion and one nucleotide deletion were observed in polyprotein precursors, 5'-UTR and 3'-UTR among the two isolates from 2010 against 2007's respectively. Two inserted nts were observed at the 98th and the 120th site in 5'-UTR respectively, and one nt was deleted at the 14th site in 3'-UTR in two 2010 genomes. Mutation aa residues were distributed mainly in VP2 (43, D → N; 153, K → R; 165, A → T; 172,R → K), VP3 (156, D → E;182,Y → N), VP1 (301,N → D), 2A (71,Y → H;75,T → S;104,F → Y;137,V → I), 2B (62,S → T), 2C (256,I → V), 3C^Pro ^(68,V → T;150,T → V) and 3D (21,S → R;42,A → V,139,K → R;147,T → A;371,V → I) regions. Comparing the mutation of CA24v nucleotides and amino acids, we found roughly 90% nucleotide mutations were synonymous mutations.

## Discussion

AHC is a common disease in China. As CVA24v and influenza A virus share the same receptor (sialic acid) [17], CVA24v is frequently associated with respiratory disease [18]. Causative agents are likely spread among people through conjunctival secretions and direct contact, but respiratory transmission may also occur [19] and may even explain the rapid and extensive spread of AHC during outbreaks [19]. The past 10 years is associated with rapid urbanization in China, with more of the population living in urban areas. High population density and poor sanitation permit these causative agents to spread quickly. Schools, particularly kindergartens and factories become key places for AHC outbreaks to develop. Several large AHC outbreaks were documented in China in 1971, 1988, 1994, 2002 and 2007 [20]. Periodic outbreaks are the result of considerable infection immunity decline after 7 years. This loss of herd immunity to the virus may have permitted the widespread transmission and periodic outbreaks [21]. Analyzing data from the AHC surveillance system, we find that most AHC outbreaks in Guangdong occur during the period from August to October in the autumn season. Mild climate and school openings in the autumn provide adaptive conditions for CA24v outbreaks.

Sex and age distribution were described in this study. In previous studies, ≦ 9 and ≧ 45 age groups were of lower risk, with children being the notably affected age group since 2007. Interestingly, the male to female ratio is higher in the ≦ 49 than in ≧ 50 age group in the two recent outbreaks. The highest ratio (1.86:1) occurred in ≦ 9 age group, and the ratio decreases with the increase of age. A similar ratio (1.9:1) was described in hand-foot-mouth disease caused by enterovirus 71 and coxsackievirus 16 [22]. This predominance in children has been observed in other enteroviral infections, in which the male-to-female ratio ranged from 1.5:1 to 2.5:1 [23,24]. These results cannot be reasonably explained at present, but may suggest susceptibility at the host genetic and immune level.

In order to identify the phylogenetic relationship of the representative strains from the large Guangdong outbreak of 2010, we amplified and sequenced the 3C^Pro ^and VP1 of EV to screen genetic material and type positive EV specimens. In this study, nucleotide sequences of the complete VP1 and partial 3C^Pro ^regions were compared, together with the prototype strain (Singapore) in 1970 and other worldwide strains. The results show the phylogenetic trees of CA24v were similar with that previously reported [13]. Due to the high recombination rates in 3C^Pro ^region and the major neutralization sites in VP1 region, both are generally used for evolution analysis, but some propose that the VP1 region is demonstrably more informative for molecular epidemiological studies than the 3C^Pro ^region. Comparing phylogenetic trees of VP1 with 3C^Pro^'s and the genome tree, we found both the VP1 and 3C^Pro ^trees could correctly determine four genotypes, but the VP1 tree was superior than the 3C^Pro^'s chronologically. This indicated that the VP1 sequence was more informative than the 3C^Pro ^sequence regarding genetic trees. In addition, the phylogenetic trees show that isolates from five cities shared one cluster and had a close relationship among them. The nucleotide distance observed within them suggests a common source.

In this study, the two outbreaks, 2007 and 2010, were compared. The scale of the 2010 outbreak is 2.28 times larger than the 2007 outbreak in population. Obviously, the 2010 outbreak curve shapes are different from 2007's. One notable difference is that one large peak was observed in the ≦ 9 age group, which had been the highest risk population in the 2010 outbreak. The absence of protective antibodies against enterovirus in young children may result in higher risk, which can be observed in other enterovirus diseases as well [22].

The 5'-and 3'-UTR of the enterovirus genome contribute to the replication of the virus RNA [25]. The 5'-UTR of the enterovirus RNA contains an *ori*L (nt 1-90) and an IRES (nt 91-745), which directs the initiation of translation in a cap-independent manner [26]. In this study, we found that two inserted nucleotides resided at the 98th and 120th sites of the 5'-UTR of two 2010 genomes. Curiously, the insertion is not found in other VA24v genomes. As the two inserted nucleotides were located in the IRES of CA24v, we infer the two insertions may have an effect on the initiation of translation of CA24v. Coincidentally, one nt deletion occurred at the 14th of 3'-UTR, and the deletion is only found in two 2010 genomes. The 3'-UTR of the enterovirus genome contains virus oriR [25]. Since 3'-UTR is a strictly *cis*-acting element that needs to make contact with the 5'-oriL [25], we propose the deletion may work in concert with the insertion of the 5'-URT.

Immunologic barriers against CA24v are usually formed among the infected population after a large outbreak or pandemic, which will hamper the viruses' ability to quickly spread among population. To escape from neutralization, like influenza A, CVA24v will normally rapidly change its' antigen epitope, which results in a CA24v outbreak in a short time in the same region. The high evolutionary rate has been estimated to be 3.0-3.7 × 10^-3 ^substitutions per site per year, 30 years after the emergence of the CVA24v [27], which is similar to influenza A (2.4-3.4 × 10^-3^) [28]. To further elucidate the CA24v antigen epitope variation, genome amino acid sequences from the 2010 outbreak strains were compared with 2007's. One, two and four amino acid changes were found in VP1, VP3 and VP2 respectively. In enterovirus, VP1, VP2, and VP3 proteins are located at the surface of the viral capsid and are exposed to immune pressure [29]. Major neutralization sites reside in the VP1, VP2 and VP3 proteins, but VP1 contains most of the neutralizing epitopes [30]. Previous studies show most antigen sites induced neutralizing antibodies reside in N-terminal [31], few sites were found at C terminus of VP1. But the C-terminal region (293-302 C-terminal) of the VP1 protein has also been shown to be highly antigenic by using peptide scanning techniques in CA9 [32]. An aa variation at the C terminus of VP1 was observed (301,N → D) in two 2010 outbreak strains. Aspartic acid (D) and asparagine (N) have a similar space structure, but different hydrophilicity. Two hydrophilicity and one hydrophobicity aa change were observed on VP2 (43 D → N; 165 A → T) and the VP3 (182 Y → N) regions as well. Hydrophilic aa are usually exposed to the surface of the capsid protein and form antigen epitope sites. In contrast, hydrophobic aa resides inside of the capsid protein. This indicated that these aa substitution might have an effect on capsid protein secondary structure and antigen epitopes, eventually caused escape mutation, promoting an AHC outbreak in a relatively short time span.

## Conclusions

We described the 2010 Guangdong outbreak of AHC, and identified the likely etiological agent to be CA24v. We characterized the full genome of 4 CA24v strains from 2010 and 2007. Sequence comparison, phylogenetic analyses, and evolutionary studies reveal that CA24v throughout the world has been divided into 4 genotypes. The CA24v causing the 2010 Guangdong outbreak belongs to genotype IV, and VP1 is more informative than 3C^Pro ^for describing molecular epidemiology. This study also confirms significant variations of multiple aa and nt in the 2010 genome, which helps to infer that accelerative virus change may have promoted the AHC outbreak in 2010.

## Materials and methods

### Sample collection and virus isolation

A total of 56 conjunctival swabs collected from 56 AHC patients were processed immediately by using 4 ml Hanks media containing penicillin (1,000 u/ml) and streptomycin (1000 μg/ml) for 4 h. They were then cultured in fresh monolayers of the HEp-2 cells [8]. The cell line was maintained in the medium supplemented with 10% FBS. When the cells in monolayer presented 70% of confluence, the medium was discarded and 0.2 ml of conjunctival sample was added to a 24-well culture plate. Specimens were allowed to adsorb for 1 h at 36°C before adding 1 ml of fresh MM plus 2% FBS to each well. The cultures were incubated at 36°C and observed daily for CPE for 7 days, with the medium replaced every 4 days. Two blind passages were performed when no CPE was observed. To compare the genome of CA24v, 2 isolates from 2007 were used.

### Ethics Statement

Use of conjunctival swab, which was collected for this study, was approved by the Ethical Committee for Centers for Disease Control and Prevention, and written informed consent was obtained from the study participants.

### RNA extraction and RT-PCR

RNA was extracted from virus culture supernatant with a QIAamp MinElute Virus Spin kit (QIAGEN Inc., Valencia, Calif)) according to the manufacture's instruction manual.

Eight-pair primers were designed according to CA24v strain sequences (DQ443002) from Singapore. A one-step RT-PCR amplification reaction was performed by using the SuperScript TM III OneStep RT-PCR with Platinum Taq (Invitrogen USA). The reaction system consisted of 2 × Reaction Mix (a buffer containing 0.4 mM of each dNTP, and 2.4 mM MgSO4), 0.5 μl SuperScript III RT/Platinum Taq High Fidelity Enzyme Mix, and 0.4 μM specific primers CVA24v-S and CVA24v-A for VP1, specific primers 3C-1 and 3C-2 for 3C region or 0.5 μM for other specific primers (Table 1) for other regions of the genome. 5 μl of extracted RNA was added to a final volume of 25 μl. The cycling conditions for the 10 RT-PCRs for the genome were: an initial cycle at 45°C 10 min, 50°C for 20 min and 94°C for 2 min; followed by 35 cycles at 94°C for 30 s, 45°C 30 s (increase in increments of 0.3°C for 1 s each up to 55°C) and 68°C for 1 min; and a final incubation at 68°C for 10 min. The band of PCR amplicons visualized after electrophoresis were subsequently excised from 1% agarose gel, and purified by use of a QIAGEN gel extraction kit (QIAGEN, Germany).

**Table 1:** Primer sequences used in this study

Primers	Regions	Nucleotide sequences (5'→3')	Nucleotide position	Amplicons (bp)	References
CA24-1F	5'-UTR	GAAATTAAAACAGCTCTGGGGTTGTTCC	1~820	820	This study
CA24-1R	VP4	TGGATCCGCCAGTTGCCACATTAG			
CA24-2F	5'-UTR	GGCAACACTATTACAATGGGTGCC	736~1620	885	This study
CA24-2R	VP2	GGCCATACAGTCTATTGCTAGGGAG			
CA24-3F	vp2	CGAATAATTCGGCAACTCTTGTGCTG	1558~2523	965	This study
CA24-3R	vp1	AGTAATAACGGTGTCAATGGTCTCC			
CVA24v-S	VP3	GTGAGTGCTTGCCCAGATTT	2407~3438	1032	Ding et al. 2009
CVA24v-A	2a	ATACACCGCCATGTTCTGGT			
CA24-5F	vp1	TAAGGTGTTGGTGTCCTAGACCG	3292-4197	905	This study
CA24-5R	2c	CCCTTTGGCAGCATTACAGGC			
CA24-6F	2b	GCGACTGTTCACCCTGGC	4072-4853	781	This study
CA24-6R	2c	TCGCTGTGTGAGACTGTTGGAG			
CA24-7F	2c	GCAACGACATGAAGCTGTTCTGTC	4696-5322	626	This study
CA24-7R	3a	CAAAACAGTCATGGCCCTGTTCAG			
3C-1	3a	AAAGGGATGGATCGTCAAGC	5250~6181	932	Wu et al.2008
3C-2	3d	TAGCCTCTTCAAAGTCTGTC			
CA24-9F	3d	GAGCCCAGTGTCTTCCATTGTG	6084-6748	664	This study
CA24-9R	3d	CGAACCATGCAGGACTGAGTGA			
CA24-10F	3d	CCAGGCGTAGTGACAGGATCAG	6612-7461	849	This study
CA24-10R	3'-UTR	CCGAATTAAAGAAAAATTTACCCCTACAAC			

### Sequencing and genetic analysis

Nucleotide sequencing reactions were performed with a BigDye terminator v3.1 cycle sequencing kit (Applied Biosystems). They were subjected to the initial denaturation at 96°C for 2 min and 30 cycles consisting of 96°C for 10 s, 50°C for 5 s, and 60°C for 4 min in a Gene Amp PCR system 2700 (Applied Biosystems). The products labeled by fluorescence were purified by use of the illustra Autoseq G-50 kit (Amersham Biosciences, UK^Q10^) and applied to ABI 3100 Genetic Analyzer (Applied Biosystems).

For molecular typing, nucleotide sequences for PCR amplicons by both CVA24v-S and CVA24v-A covering the entire VP1 region and 3C-1 and 3C-2 for 3C region, were used and compared with the sequence of EH24/70 strain.

To identify respective divergence and infer the genetic relationship among the isolates, the nucleotide sequences were analyzed by using MEGA software version 5.0. Phylogenetic trees were constructed by a neighbor-joining method after 1000 bootstrapping [33].

### Nucleotide sequence accession numbers

The VP1 and 3C sequences from this study are available in GenBank with the following accession numbers: JF742580-JF742595, and JF742560-JF742575 We also sequenced the genome of CA24v isolates from four isolations. GenBank accession numbers of these sequences are JF742576-JF742579.

## Abbreviations

AHC: Acute hemorrhagic conjunctivitis; CA24v: Variant of coxsackievirus A24; EV70: Enterovirus 70; CPE: Cytopathic effect; FBS: Fetal bovine serum AA Amino acid; NDSIMS: National disease supervision information management system; UTR: Untranslated region; IRES: Internal ribosome entry sites; Nt: Nucleotide.

## Competing interests

The authors declare that they have no competing interests.

## Authors' contributions

WD carried out genetic analysis, drafted the manuscript. ZH_Y _performed whole-genome sequencing, participated in the study design. GX carried out viral isolation. LL carried out viral isolation. MY participated in sample collection. ZH_R _participated in sample collection. ZH participated in sample collection. ZH_Q _performed the sequence alignment. FL performed the sequence alignment. LC participated in whole-genome sequencing. LH helped to draft the manuscript. KC participated in the design of the study. MC helped editing of the manuscript. HY helped editing. All authors read and approved the final manuscript.
